# The complete mitochondrial genome and phylogenetic analysis of *Batillaria zonalis* (Gastropoda: Batillariidae)

**DOI:** 10.1080/23802359.2020.1772139

**Published:** 2020-06-01

**Authors:** Chengrui Yan, Jiantong Feng, Yingying Ye, Jiji Li, Baoying Guo

**Affiliations:** a National Engineering Research Center for Marine Aquaculture, Zhejiang Ocean University, Zhoushan, China; b Key Laboratory of Informatization of Habitat Monitoring and Fishery Resource Conservation Research in the East China Sea of Zhejiang Province, Zhejiang Ocean University, Zhoushan, China

**Keywords:** *Batillaria zonalis*, mitochondria genome, phylogenetic, Illumina

## Abstract

We determined the complete mitochondrial genome of *Batillaria zonalis.* The *B. zonalis* mitochondrial genome is 15748 bp in length, comprising 13 protein-coding genes, 22 transfer RNA genes, and two ribosomal RNA genes. The nucleotide composition for *B. zonalis* is 17.71% of C, 16.74% of G, 34.99% of T, and 30.3% of A. In 13 protein-coding genes, all genes start with ATG. For the stop codon, the cox2 gene stops with TTC, the cytb, nad1, nad2 genes stop with TAG, the other nine genes are with TAA. Of these 37 genes identified, nine protein-coding genes and six transfer RNA genes are encoded on the heavy strand and the other genes on the light strand. The phylogenetic tree was constructed based on 13 protein-coding genes of the *B. zonalis* and other 19 Gastropoda species, *Sepia latimanus* as outgroup using the Neighbour-joining method. The result showed that *B. zonalis* is most closely related to the *Tylomelania sarasinorum* in Cerithioidea. We believe that this result will be helpful for the study of population genetic and phylogenetic analysis of the family Batillariidae.

*Batillaria zonalis* (Bruguière, 1792) belong to the family Batillariidae is an intertidal gastropod. The shell is mostly conical, and sallow or brown color. *Batillaria zonalis* lives in the mudflats of the high and the middle tide in the intertidal zone. It distributed in the southern part of Okinawa Island in Japan and north and south coastal areas of China Sea (Kamimura 2000). In the Japanese Islands, *B. zonalis* are now threatened and treated as an endangered species (Wada et al. 1996). At present, there is no research on the mitochondrial genome of *B. zonalis*.

This is the first report of a complete mitochondrial genome sequence of *B. zonalis.* The specimen of *B. zonalis* was collected in Dalian, Liaoning province, China (121.44°E, 39.01°N), identified by morphology and stored in a refrigerator at −20 °C in Zhejiang Engineering Research Center for Mariculture and Fishery Enhancement Museum (Accession number: BZ20191025). The total DNA extraction was utilized the salting-out method (Aljanabi and Martinez 1997) with the muscle. The genomic DNA was prepared in 400 bp paired-end libraries, and The Illumina HiSeq X Ten platform was using total genomic DNA to sequence the mitochondrial genome. All the data was available and enumerated to the Microsoft oneDrive database (https://1drv.ms/w/s!ArF1Al5lLW_VaxWz_2PJzvwa6EU?e=hjWHCk).

The *B. zonalis* mitochondrial genome is 15,748 bp in length (GenBank accession number: MT363252), comprising 13 protein-coding genes, 22 transfer RNA genes, and two ribosomal RNA genes. The nucleotide composition for *B. zonalis* is 17.71% of C, 16.74% of G, 34.99% of T, and 30.3% of A. In 13 protein-coding genes, all genes start with ATG. For the stop codon, the cox2 gene stops with TTC, the nad5 gene stops with TTA, the nad2 gene stops with AAG, the nad4l, nad4, nad1 genes stop with TAG, the other seven genes are with TAA. Of these 37 genes identified, nine protein-coding genes and six transfer RNA genes are encoded on the heavy strand and the other genes on the light strand. The 12S rRNA is between the tRNA^Thr^ and tRNA^Ser^, and the 16S rRNA is between the tRNA^Val^ and tRNA^Leu^.

The phylogenetic tree was constructed based on 13 protein-coding genes of the *B. zonalis* and other 19 Gastropoda species, *Sepia latimanus* as outgroup using the Neighbour-joining method (Saitou and Nei 1987) by the program Phylip (Felsenstein 1989). The tree showed that the *B. zonalis* is closely related to the *Tylomelania sarasinorum* in Cerithioidea, close to Cypraeidae and Architaenioglossa (Figure 1). We believe that this result will be one supplement of the genome information in mitochondrial of the family Batillariidae and facilitate the study on population genetic.

**Figure 1.**
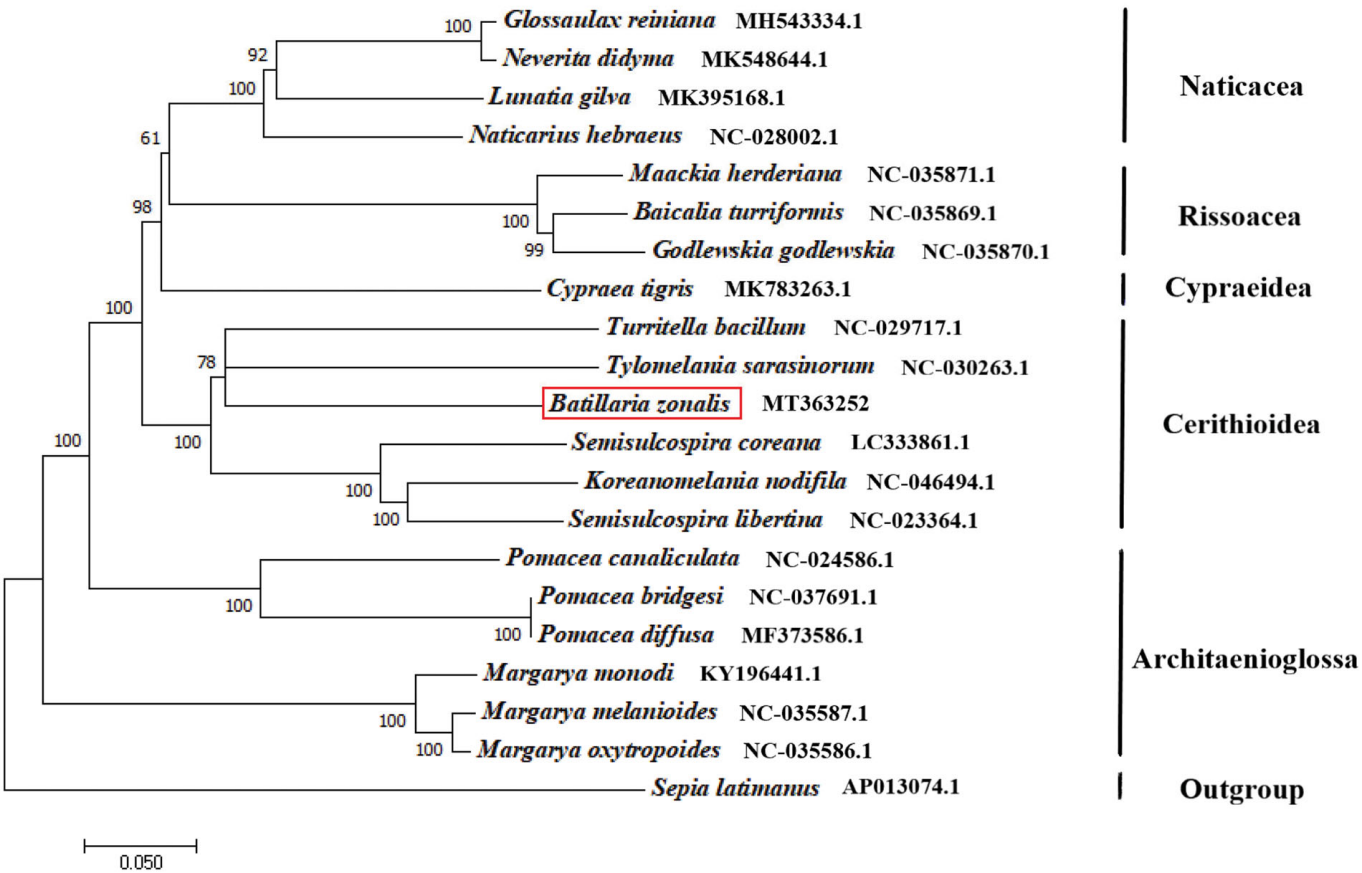
The *NJ* phylogenetic tree for *Batillaria zonalis* and other species based on 13 protein-coding genes.

## Data Availability

The data that support the findings of this study are openly available in Microsoft OneDrive at https://1drv.ms/w/s!ArF1Al5lLW_VaxWz_2PJzvwa6EU?e=hjWHCk; and in Genbank, reference number: MT363252.
